# Supplementary material for: Modeling glioblastoma heterogeneity as a dynamic network of cell states
Source: Mol Syst Biol. 2021 Sep 16;17(9):e10105. doi: 10.15252/msb.202010105 (PMC8444284; doi:10.15252/msb.202010105)
Supplement: Supplementary file 5 — Source Data for Figure 3 [file MSB-17-e10105-s001.zip › Figure3A_sourcedata/GSEA_3065/nosigdb_state6.GseaPreranked.1622718443993/OLSEN_SCPLIKE.html]

Details for gene set OLSEN\_SCPLIKE[GSEA]

|  || Dataset | state6 |
| Phenotype | NoPhenotypeAvailable |
| Upregulated in class | na\_neg |
| GeneSet | OLSEN\_SCPLIKE |
| Enrichment Score (ES) | -0.5819575 |
| Normalized Enrichment Score (NES) | -2.2376478 |
| Nominal p-value | 0.0 |
| FDR q-value | 0.0 |
| FWER p-Value | 0.0 |
Table: GSEA Results Summary

  

Fig 1: Enrichment plot: OLSEN\_SCPLIKE      
 Profile of the Running ES Score & Positions of GeneSet Members on the Rank Ordered List

  

| PROBE | GENE SYMBOL | GENE\_TITLE | RANK IN GENE LIST | RANK METRIC SCORE | RUNNING ES | CORE ENRICHMENT || 1 | BTG1 |  |  | 4 | 0.348 | 0.0335 | No |
| 2 | S100A16 |  |  | 120 | 0.204 | 0.0402 | No |
| 3 | PNRC1 |  |  | 123 | 0.202 | 0.0597 | No |
| 4 | CRYAB |  |  | 173 | 0.184 | 0.0721 | No |
| 5 | ZFP36L1 |  |  | 266 | 0.159 | 0.0770 | No |
| 6 | GAS7 |  |  | 268 | 0.157 | 0.0923 | No |
| 7 | TOMM7 |  |  | 310 | 0.149 | 0.1022 | No |
| 8 | S100B |  |  | 391 | 0.137 | 0.1064 | No |
| 9 | RHOC |  |  | 794 | 0.099 | 0.0699 | No |
| 10 | QKI |  |  | 820 | 0.097 | 0.0765 | No |
| 11 | CTNNAL1 |  |  | 1078 | 0.082 | 0.0550 | No |
| 12 | MT2A |  |  | 1097 | 0.081 | 0.0608 | No |
| 13 | EDNRB |  |  | 1266 | 0.073 | 0.0487 | No |
| 14 | IFITM3 |  |  | 1398 | 0.068 | 0.0403 | No |
| 15 | PMP2 |  |  | 1515 | 0.063 | 0.0331 | No |
| 16 | RASSF4 |  |  | 1591 | 0.061 | 0.0305 | No |
| 17 | LIMA1 |  |  | 1702 | 0.057 | 0.0234 | No |
| 18 | OLFML2A |  |  | 1756 | 0.055 | 0.0227 | No |
| 19 | MEF2C |  |  | 1785 | 0.054 | 0.0247 | No |
| 20 | ANXA5 |  |  | 1978 | 0.048 | 0.0074 | No |
| 21 | CLIC4 |  |  | 2035 | 0.047 | 0.0056 | No |
| 22 | SELENOM |  |  | 2281 | 0.039 | -0.0187 | No |
| 23 | PMEPA1 |  |  | 2388 | 0.036 | -0.0273 | No |
| 24 | S100A11 |  |  | 2523 | 0.033 | -0.0395 | No |
| 25 | AP1S2 |  |  | 2699 | 0.029 | -0.0567 | No |
| 26 | AHR |  |  | 2711 | 0.029 | -0.0552 | No |
| 27 | VAMP5 |  |  | 2750 | 0.028 | -0.0568 | No |
| 28 | VIM |  |  | 2831 | 0.026 | -0.0635 | No |
| 29 | FN1 |  |  | 2839 | 0.026 | -0.0618 | No |
| 30 | ARPC1B |  |  | 3027 | 0.021 | -0.0811 | No |
| 31 | CNN3 |  |  | 3415 | 0.013 | -0.1243 | No |
| 32 | SPTBN1 |  |  | 4188 | -0.003 | -0.2125 | No |
| 33 | S100A6 |  |  | 4326 | -0.005 | -0.2277 | No |
| 34 | ERBB3 |  |  | 4525 | -0.009 | -0.2495 | No |
| 35 | ZEB2 |  |  | 4735 | -0.013 | -0.2722 | No |
| 36 | TIMP3 |  |  | 5017 | -0.019 | -0.3026 | No |
| 37 | S100A10 |  |  | 5285 | -0.024 | -0.3309 | No |
| 38 | IFITM2 |  |  | 5741 | -0.033 | -0.3798 | No |
| 39 | CST3 |  |  | 5892 | -0.036 | -0.3935 | No |
| 40 | IFI6 |  |  | 6498 | -0.050 | -0.4580 | No |
| 41 | CD99 |  |  | 6572 | -0.052 | -0.4613 | No |
| 42 | PSAP |  |  | 6739 | -0.057 | -0.4748 | No |
| 43 | IGFBP5 |  |  | 7011 | -0.065 | -0.4995 | No |
| 44 | IFI16 |  |  | 7041 | -0.066 | -0.4964 | No |
| 45 | PLP1 |  |  | 7138 | -0.069 | -0.5006 | No |
| 46 | CTSD |  |  | 7147 | -0.069 | -0.4948 | No |
| 47 | IGFBP7 |  |  | 7318 | -0.075 | -0.5070 | No |
| 48 | LGALS3 |  |  | 7566 | -0.084 | -0.5271 | No |
| 49 | ITGA6 |  |  | 8043 | -0.110 | -0.5709 | Yes |
| 50 | PRNP |  |  | 8140 | -0.117 | -0.5705 | Yes |
| 51 | CAVIN3 |  |  | 8162 | -0.118 | -0.5614 | Yes |
| 52 | ITGB8 |  |  | 8195 | -0.121 | -0.5532 | Yes |
| 53 | SPARC |  |  | 8303 | -0.132 | -0.5526 | Yes |
| 54 | COL18A1 |  |  | 8322 | -0.134 | -0.5415 | Yes |
| 55 | GSN |  |  | 8339 | -0.137 | -0.5300 | Yes |
| 56 | SEMA3B |  |  | 8382 | -0.144 | -0.5207 | Yes |
| 57 | CD59 |  |  | 8508 | -0.163 | -0.5191 | Yes |
| 58 | COL4A2 |  |  | 8525 | -0.168 | -0.5046 | Yes |
| 59 | PMP22 |  |  | 8560 | -0.175 | -0.4914 | Yes |
| 60 | NTM |  |  | 8612 | -0.196 | -0.4781 | Yes |
| 61 | GPM6B |  |  | 8632 | -0.203 | -0.4604 | Yes |
| 62 | DST |  |  | 8644 | -0.211 | -0.4410 | Yes |
| 63 | ANXA1 |  |  | 8651 | -0.213 | -0.4209 | Yes |
| 64 | COL4A1 |  |  | 8661 | -0.218 | -0.4006 | Yes |
| 65 | ITM2B |  |  | 8705 | -0.242 | -0.3819 | Yes |
| 66 | B2M |  |  | 8718 | -0.258 | -0.3581 | Yes |
| 67 | ANXA2 |  |  | 8741 | -0.279 | -0.3334 | Yes |
| 68 | TIMP1 |  |  | 8751 | -0.290 | -0.3061 | Yes |
| 69 | APP |  |  | 8758 | -0.300 | -0.2775 | Yes |
| 70 | VMP1 |  |  | 8760 | -0.302 | -0.2481 | Yes |
| 71 | CD9 |  |  | 8762 | -0.303 | -0.2186 | Yes |
| 72 | PLAT |  |  | 8772 | -0.327 | -0.1877 | Yes |
| 73 | NEAT1 |  |  | 8775 | -0.334 | -0.1553 | Yes |
| 74 | CD63 |  |  | 8776 | -0.335 | -0.1226 | Yes |
| 75 | ITGB1 |  |  | 8777 | -0.335 | -0.0899 | Yes |
| 76 | PTPRZ1 |  |  | 8783 | -0.360 | -0.0554 | Yes |
| 77 | MALAT1 |  |  | 8796 | -0.581 | 0.0000 | Yes |
Table: GSEA details [plain text format]

  

Fig 2: OLSEN\_SCPLIKE: Random ES distribution      
 Gene set null distribution of ES for **OLSEN\_SCPLIKE**
